# High Glucose and Hypoxia-Mediated Damage to Human Brain Microvessel Endothelial Cells Induces an Altered, Pro-Inflammatory Phenotype in BV-2 Microglia In Vitro

**DOI:** 10.1007/s10571-020-00987-z

**Published:** 2020-11-02

**Authors:** Jaclyn Iannucci, Haripriya Vittal Rao, Paula Grammas

**Affiliations:** 1 The George & Anne Ryan Institute for Neuroscience, University of Rhode Island, 130 Flagg Road, Kingston, RI 02881 United States; 2 Department of Biomedical and Pharmaceutical Sciences, College of Pharmacy, University of Rhode Island, Kingston, RI 02881 USA; 3 Wake Forest Baptist Medical Center, Winston-Salem, Wake Forest, NC 27101 USA

**Keywords:** Vascular, Inflammation, Endothelial, Microglia, Alzheimer’s disease

## Abstract

Diabetes is strongly linked to the development of Alzheimer’s disease (AD), though the mechanisms for this enhanced risk are unclear. Because vascular inflammation is a consistent feature of both diabetes and AD, the cerebral microcirculation could be a key target for the effects of diabetes in the brain. The goal of this study is to explore whether brain endothelial cells, injured by diabetes-related insults, glucose and hypoxia, can affect inflammatory and activation processes in microglia in vitro. Human brain microvascular endothelial cells (HBMVECs) were either treated with 5 mM glucose (control), 30 mM glucose (high glucose), exposed to hypoxia, or exposed to hypoxia plus high glucose. HBMVEC-conditioned medium was then used to treat BV-2 microglia. Alterations in microglia phenotype were assessed through measurement of nitric oxide (NO), cytokine production, microglial activation state markers, and microglial phagocytosis. HBMVECs were injured by exposure to glucose and/or hypoxia, as assessed by release of LDH, interleukin (IL)-1β, and reactive oxygen species (ROS). HBMVECs injured by glucose and hypoxia induced increases in microglial production of NO, tumor necrosis factor-α (TNFα) and matrix metalloproteinase (MMP)-9. Injured HBMVECs significantly increased microglial expression of CD11c and CLEC7A, and decreased expression of the homeostatic marker P2RY12. Finally, bead uptake by BV-2 cells, an index of phagocytic ability, was elevated by conditioned media from injured HBMVECs. The demonstration that injury to brain endothelial cells by diabetic-associated insults, glucose and hypoxia, promotes microglial inflammation supports the idea that the cerebral microcirculation is a critical locus for the deleterious effects of diabetes in the AD brain.

## Background

Alzheimer’s disease (AD) is a neurodegenerative disorder characterized by progressive loss of cognitive function, leading to dementia and death (Lane et al. 2018). AD is a complex, multifactorial disorder where disparate processes likely converge to produce neuronal injury. Cardiovascular risk factors (CVRFs) are important risk factors for the development of AD (Hofman et al. 1997; Helzner et al. 2009; Dickstein et al. 2010; Tolppanen et al. 2012; Yaffe et al. 2014). In particular, individuals with type 2 diabetes mellitus (T2D) have a greater than 50% increased risk for developing AD ( Ott et al. 1999; Janson et al. 2004; Gudala et al. 2013). In the periphery, diabetes and other CVRFs are potent drivers of inflammation (Lontchi-Yimagou et al. 2013). Because neuroinflammation is a consistent feature of AD, and other neurodegenerative diseases, inflammation could be a key target for the effects of diabetes in the brain.

Sustained hyperglycemia, a pathognomonic feature of diabetes, has been shown to drive endothelial cell dysfunction in the periphery via a number of signaling mechanisms, including increased expression of reactive oxygen species (ROS) and inflammatory proteins (Blake and Trounce 2014; Eelen et al. 2015; Stefano et al. 2016). Microvascular endothelial cells at the blood-brain interface are the cells in the brain most directly exposed to the deleterious effects of high glucose levels. In this regard, both in vitro models utilizing high glucose conditions and in vivo experiments with diabetic animals have shown compromised blood-brain barrier (BBB) integrity and increases in inflammatory proteins, including tumor necrosis factor-α (TNFα), and matrix metalloproteinase (MMP)-9 (Bogush et al. 2017; Rom et al. 2019) .

There is a similar robust elevation in the expression of inflammatory mediators in the cerebral microcirculation in AD. AD brain endothelial cells express high levels of inflammatory adhesion molecules, such as monocyte chemoattractant protein-1 (MCP-1), intercellular adhesion molecule-1 (ICAM-1), and cationic antimicrobial protein 37 kDa (CAP37) (Frohman et al. 1991; Pereira et al. 1996; Grammas and Ovase 2001). Additionally, AD brain microvessels release significantly higher levels of nitric oxide (NO), thrombin, TNFα, transforming growth factor-β (TGF-β), interleukin (IL) IL-1β, IL-6, IL-8, and MMPs compared to age-matched controls (Dorheim et al. 1994; Grammas and Ovase 2001; Grammas and Ovase 2002; Thirumangalakudi et al. 2006). Because the inflamed/damaged cerebral endothelium is a highly synthetic interface that produces numerous bioactive factors, it is likely that endothelial-derived products will have critical effects on neighboring cells of the neurovascular unit (NVU) (McConnell et al. 2017). The functional interaction of NVU cells through bidirectional cell-cell signaling is critical for maintaining cerebral blood flow and regulating physiologic processes in the CNS (Muoio et al. 2014). Microglia-endothelial cell cross-talk can influence BBB function and permeability (Presta et al. 2018). For example, microglia activated by treatment with LPS can induce alterations in brain endothelial cell morphology and function (Dudvarski Stankovic et al. 2016), but less is known about endothelial cell effects on microglia.

Microglia, the brain’s resident immune cells, are key drivers of the neuroinflammatory response in AD and other neurodegenerative disorders (Akiyama et al. 2000; Calsolaro and Edison 2016). Once described as existing in a quiescent or resting state, microglia are now understood to be dynamic cells that are constantly surveying their microenvironment for injury-inducing mediators (Hanisch and Kettenmann 2007). Microglia are believed to assume a diversity of phenotypes in response to noxious extracellular stimuli (Chitnis and Weiner 2017; Hickman et al. 2018). Multiple groups have identified a disease associated microglia (DAM) or microglial neurodegenerative phenotype (MGnD), which is present in AD (Keren-Shaul et al. 2017; Krasemann et al. 2017). These DAM show decreased expression of homeostatic microglia genes, including p2ry12/p2ry13 and tmem119, and increased expression of pro-inflammatory and AD-associated genes, including apoe, clec7a, and cd11c. Additionally, proliferation and activation of microglia in the brain, concentrated around amyloid plaques, is a prominent feature of AD (Matsuoka et al. 2001; Fakhoury 2018), and a number of recently identified AD-risk genes are related to regulation of microglia and the innate immune system, including triggering receptor expressed on myeloid cells 2 (TREM2) (Jones et al. 2010; Guerreiro et al. 2013; Jonsson et al. 2013; Lambert et al. 2013). These findings suggest that shifts in microglia function and phenotype are a key part of the pathological development of AD.

The goal of this study is to explore whether injured brain endothelial cells can affect inflammatory and activation processes in microglia. In our in vitro system, cultured brain endothelial cells are injured by exposure to stimuli that are important in the development of diabetic pathology, namely high glucose and hypoxia. The ability of conditioned media from these injured endothelial cells to alter microglial inflammatory proteins, activation markers, and phagocytic activity is assessed in order to test the hypothesis that brain endothelial cell injury will promote pro-inflammatory activation of microglia.

## Methods

### Cell Culture and Other Reagents

Human brain microvascular endothelial cells (HBMVECs- ACBRI 376) and complete medium with recombinant growth factors (4Z0-500) were purchased from Cell Systems (Kirkland, WA, USA). Murine immortalized microglia (BV-2) were kindly provided by Dr. Navindra Seeram (University of Rhode Island, Kingston, RI, USA). Low-glucose Dulbecco’s Modified Eagle Medium (DMEM), cell culture grade D-glucose (glucose), antibiotic/antimycotic (Ab/Am), Fetal Bovine Serum (FBS), and Bovine Serum Albumin (BSA) were purchased from Sigma Aldrich (St. Louis, MO, USA).

### Culture and Treatment of HBMVECs and BV-2

HBMVECs were grown to confluence in culture in complete medium in accordance with protocols described by Cell Systems and maintained at 37 °C in 5% CO_2_ in complete medium. Media was switched to low glucose (5mM) DMEM with 1% BSA for treatments. HBMVECs were incubated with low glucose (5mM) (control) or high glucose (30mM) in treatment media for 6 h. Cells were maintained in normoxia for the full 6 h, or were exposed to hypoxic (1% O_2_) conditions for the final 1 h of treatment. Following treatment, conditioned media were collected and centrifuged briefly at 1000×*g* in sterile conditions to pellet debris, and used for subsequent treatment of BV-2.

BV2 were maintained at 37 °C in 5% CO_2_ in low glucose (5mM) DMEM with 10% FBS, 1% Ab/Am, and 250 nM L-glutamine added. HBMVEC-conditioned media was used to treat BV-2 for 24 h. Conditioned media were collected following endothelial cell treatment with either 5 mM glucose (6 h) (EC-C), 30 mM glucose (6 h) (EC-G), exposure to hypoxia (1 h) (EC-H), or exposure to hypoxia plus high glucose (6 h) (EC-G+H).

### Cytotoxicity Assay

Cellular damage of HBMVECs was assessed by measuring lactate dehydrogenase (LDH) release in the conditioned medium. Following treatment, supernatant was transferred to a clear 96-well plate and total LDH was assessed using the cytotoxicity detection kit (Millipore Sigma, Burlington, MA, USA). Absorbance values were read at 490nm following incubation with supplied chromogenic dye and catalyst using a Synergy HTX multi-mode reader (Biotek Instruments, Winooski, VT, USA).

### Detection of Reactive Oxygen Species (ROS)

The production of ROS by HBMVECs was determined by a fluorescent probe, 2’,7’-Dichlorofluorescin diacetate (DCF-DA), using a previously described method with modification (Ma et al. 2017). HBMVECs were seeded in a black-walled, clear-bottom 96-well plate at a density of 100,000 cells per mL and allowed to grow for 36 h. Cells were then treated as previously described with high glucose with or without hypoxia for 6 h. Following the 6 h treatment, DCF-DA (10µM) was added to each well and incubated at 37 °C in the dark for 25 min (min). Cells were washed 3 times with phosphate buffered saline (PBS). The fluorescence signal of each well was measured at 495 nm (excitation) and 529nm (emission) using a SpectraMax M2 plate reader (Molecular Devices, Sunnyvale, CA, USA).

### Griess Assay

The production of NO was determined using the Griess reagent system. BV-2 were grown in clear 24-well plates at 100,000 cells/mL. Following treatment with HBMVEC-conditioned media for 24 h, culture media was transferred to a clear 96-well plate and total NO was assessed using the Griess reagent kit (Promega, Fitchburg, WI, USA). Absorbance values were read at 535nm using a Synergy HTX multi-mode reader (Biotek Instruments, Winooski, VT, USA).

### Western Blot

Following treatments, BV-2 cells were rinsed in ice-cold PBS and lysed in 10mM Tris-HCl, 150mM NaCl buffer containing protease inhibitors. Lysates were mixed with 40% 4× Sample Buffer (4× Laemmli Sample Buffer (BioRad, Hercules, CA, USA) with 10% 2-mercaptoethonal) and heated to 100 °C for 10 min. Samples were resolved by SDS-PAGE on 4–20% Novex gradient gels (Invitrogen, CA) transferred to nitrocellulose membranes (iBlot, Invitrogen, Carlsbad, CA, USA). Membranes were blocked in 5% BSA in Tris buffered saline (TBS) containing 0.05% Tween-20. Primary antibodies for this study include iNOS (Abcam, MA; Ab15323, 1:250), CLEC7A (Novus Biologicals, CO; NBO1-45514, 1:500), P2RY12 (Novus Biologicals, CO; NBP1-69246, 1:500), CD11c (Novus Biologicals, CO; MAB6950, 1:250), and β-actin (Santa Cruz, CA; AC-15, 1:10,000). Bound antibody was detected using InfraRed detectable secondary antibodies (LI-COR, NE; 1:10,000), using LI-COR Odyssey infrared scanner for imaging (LI-COR, Lincoln, NE, USA). Blots were analyzed using NIH ImageJ software and normalized against the housekeeper protein.

### Enzyme Linked Immunosorbent Assay (ELISA)

IL-1β was measured from HBMVEC-conditioned media, and TNFα and IL-6 were measured from BV-2 supernatant, using Enzyme Linked Immunosorbent Assay (ELISA) from Biolegend (San Diego, CA, USA; Human IL-1β cat. #437004, Mouse TNFα cat. #430904, Mouse IL-6 cat. #431304). The protocol provided by the manufacturer was followed, without modifications. At the conclusion of the assay, absorbance was detected at 570 nm and 450 nm using Synergy HTX multi-mode reader (Biotek Instruments, Winooski, VT, USA).

### Gelatin Zymography

Gelatin zymography was performed as previously described (Thirumangalakudi et al. 2007) to assess the activity of MMP-9 in BV-2 supernatant following treatments. Briefly, samples were run on 10% Gelatin Zymogram Plus Protein Gels (Invitrogen, Carlsbad, CA, USA). Following electrophoresis, gels were incubated at room temperature (RT) with zymogram renaturing buffer (Novex, Carlsbad, CA, USA) for 30 min, and then incubated with zymogram developing buffer (Novex, Carlsbad, CA, USA) for 30 min. Gels were further incubated with fresh developing buffer overnight at 37 °C. Gels were stained with Coomassie Blue R250 for 1 h, and de-stained in deionized water for 1 day. Images scanned on Epson Scanner (Epson, Long Beach, CA, USA) and analyzed using ImageJ.

### Phagocytosis Assay

Analysis of phagocytic activity of BV-2 was done by measuring uptake of fluorescently labeled beads. Protocol for the phagocytosis assay was derived from previously published works (Lucin et al. 2013; Lian et al. 2016; Cai et al. 2017; Zhang et al. 2017) with modifications. BV-2 cells were plated in 8-well chamber slides at a density of 10,000 cells per well in DMEM with 10% FBS and grown for 48 h. Cells were treated as before with HBMVEC-conditioned media for 24 h.

Preopsonized latex beads of 1 um diameter (Sigma, cat. #L2778-1ML) were prepared in PBS with 50% FBS at 10% (v/v) for 1 h at 37 °C. Beads were then further diluted 1:100 in DMEM to make a 0.01% (v/v) beads and 0.05% (v/v) FBS solution.

At the conclusion of 24 h treatment, BV-2 media was removed and 500 uL of bead solution was added to each well for 1 h at 37 °C. Beads were then removed and cells were rinsed three times with cold PBS. Cells were fixed with 4% paraformaldehyde (PFA) in PBS (200 uL per well) for 4 min at RT. PFA was removed, cells were then blocked and permeabilized with 5% horse serum and 0.3% Triton X-100 in PBS (200 uL per well) for 30 min at RT. Cells were washed again with cold PBS three times.

To image, antifade mounting solution with DAPI (Invitrogen, cat. #S36964) was added, slide was covered with a cover slip, and sealed. Beads were imaged at 575–610nm (red) and DAPI at 345–453 nm (blue) using the EVOS® FL Auto Cell Imaging System (ThermoFisher Scientific, Waltham, MA, USA). ImageJ was used to quantify red/blue in all images.

### Statistical Analysis

Data were analyzed for significance using one-way analysis of variance (ANOVA) and multiple comparisons carried out using the post-hoc Bonferroni test on GraphPad Prism (version 8.01). Data are represented as Mean +/− SEM. Groups contain *n* = 6 unless otherwise specified.

## Results

### Injury of Brain Endothelial Cells by Exposure to High Glucose Levels and Hypoxia

**Fig. 1:**
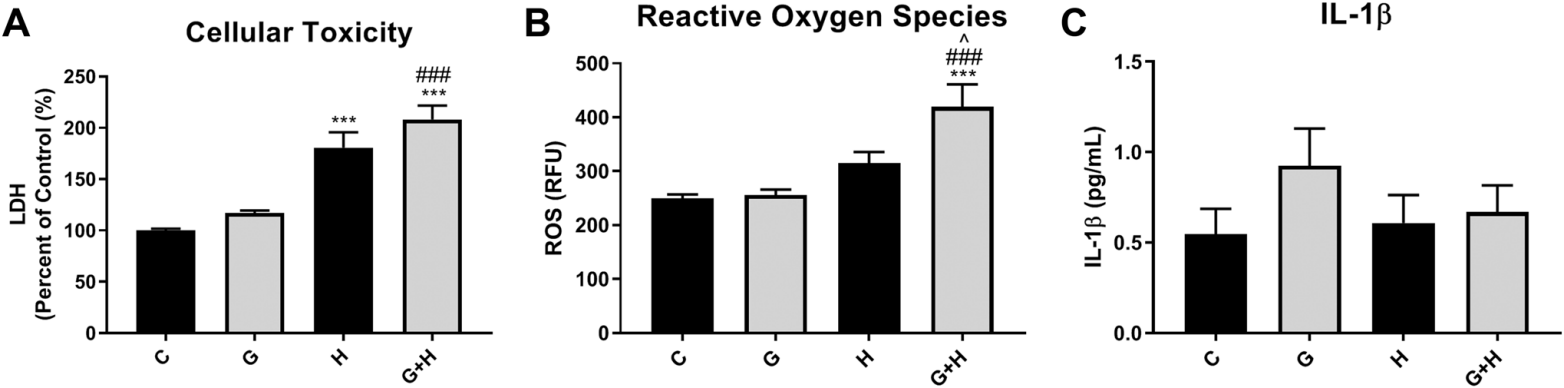
HBMVEC injury following treatment with high glucose with and without hypoxia HBMVECs were exposed to high glucose (6 h), hypoxia (1 h), or a combination of both (6 h). Cells and supernatant were collected to assess measures of cellular injury: **a** lactate dehydrogenase (LDH) release as a measure of cell toxicity, **b** reactive oxygen species (ROS) production measured with DCF-DA, represented as relative fluorescence units (RFU), and (C) IL-1β secretion in the supernatant measured by ELISA. ***p < 0.001 vs. Control, ^###^*p* < 0.001 vs. Glucose, ^*p* < 0.05 vs. Hypoxia

HBMVECs were either treated with 30 mM glucose (6 h), exposed to hypoxia (1 h), or exposed to hypoxia plus high glucose (6 h). LDH release in the supernatant was measured to estimate lethal cell injury. Treatment of cells with glucose evoked a small but not significant increase in LDH release. In contrast, hypoxia exposure significantly increased LDH release compared to untreated controls (*p* < 0.001). The level of LDH detected in cells exposed to hypoxia plus high glucose was higher than levels measured in response to hypoxia alone (Fig. 1a).

To explore indices of non-lethal injury the generation of ROS and release of the inflammatory mediator IL-1β were assessed. HBMVECs analyzed using the fluorescent probe DCF-DA to measure ROS showed that glucose treatment did not alter ROS production compared to control cells, while hypoxia exposure increased ROS levels relative to control, but the increase was not significant. HBMVECs exposed to both glucose and hypoxia showed significantly increased production of ROS compared to control (*p* < 0.001), glucose (*p* < 0.001), or hypoxia (*p* < 0.05) (Fig. 1b). Measurement of IL-1β levels in supernatant by ELISA indicated that treatment with glucose increased IL-1β levels, but the increase was not significant (*p* = 0.11), while hypoxia exposure did not affect IL-1β levels. Interestingly, the combination of glucose treatment and hypoxia exposure appeared to mitigate the increase evoked by glucose alone (Fig. 1c).

### Injured Brain Endothelial Cells Increase Reactive Nitrogen Species Production in BV-2 Cells

**Fig. 2:**
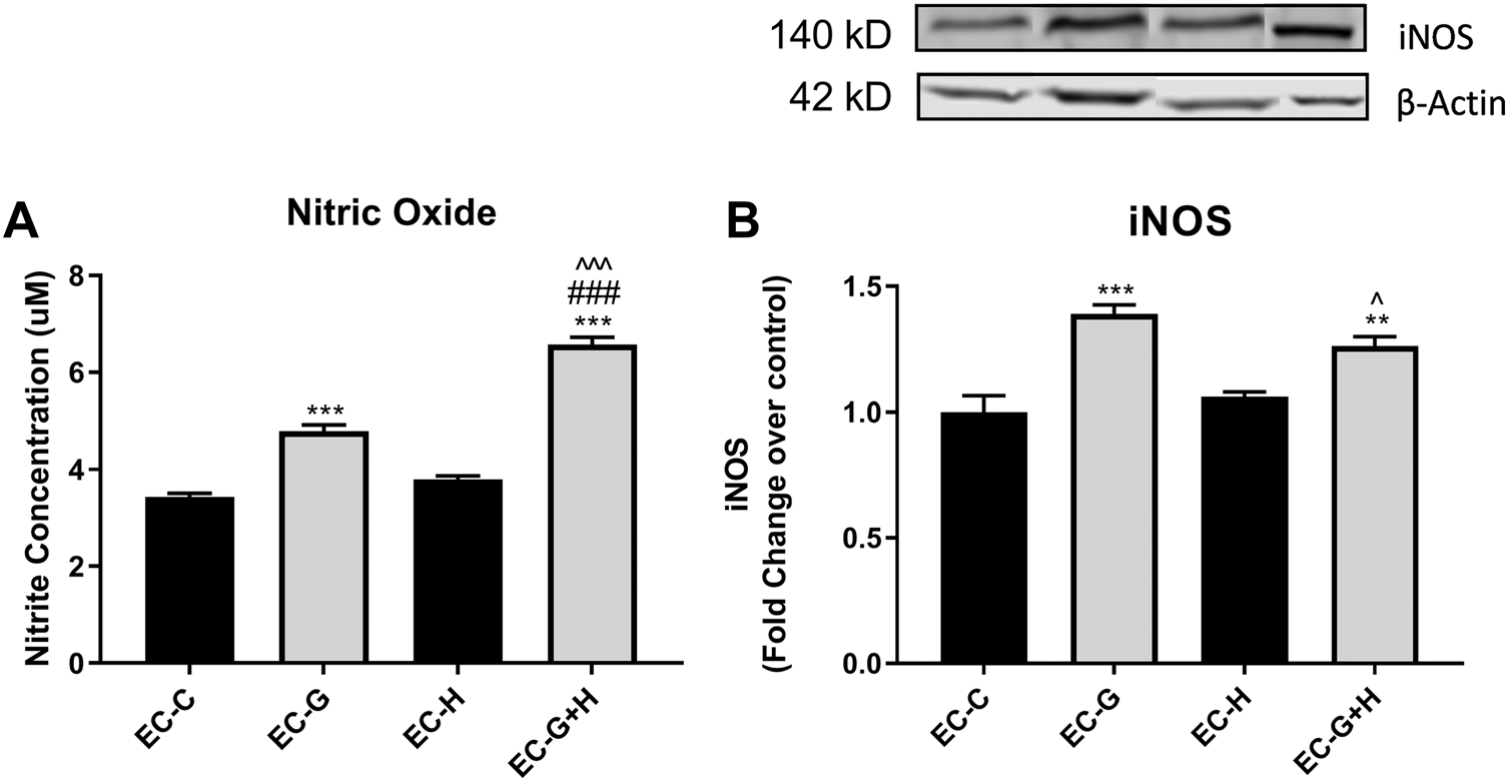
HBMVEC injury induces increased nitric oxide production by BV-2 microglia Conditioned media from injured HBMVECs was used to treat BV-2 microglia for 24 h. Supernatant and cell lysate were collected following treatments and used to measure (**a**) Nitric Oxide (NO) in the supernatant using Griess reagent (*n* = 12 per group) and (**b**) expression of iNOS in the lysate by western blot. **p < 0.01 vs. Control, ***p < 0.001 vs. Control, ^###^*p* < 0.001 vs. Glucose, ^*p* < 0.05 vs. Hypoxia, ^^^*p* < 0.001 vs. Hypoxia.

To determine whether injured endothelial cells can affect reactive nitrogen species (RNS) generation in microglia, the microglial cell line BV-2 was treated with HBMVEC-conditioned media for 24 h. The Griess assay was used to assess NO production in the BV-2 supernatant (Fig. 2a) and inducible nitric oxide synthase (iNOS) levels in BV-2 lysates were analyzed by western blot (Fig. 2b).

BV-2 cells exposed to conditioned media from glucose-treated endothelial cell cultures (EC-G) showed a significant increase in both NO production (*p* < 0.001) (Fig. 2a) and iNOS expression (*p* < 0.001) (Fig. 2b) compared to BV-2 cells incubated with untreated endothelial cell conditioned media. In contrast, treatment of BV-2 cells with conditioned media from hypoxia-exposed endothelial cells (EC-H) did not affect either NO production or iNOS levels (Fig. 2a, b). Treatment of BV-2 cells with conditioned media from endothelial cells exposed to both glucose and hypoxia (EC-G+H) significantly increased NO production compared to BV-2 cells exposed to conditioned media from only glucose-treated endothelial cell cultures (*p* < 0.001). There was no significant difference in BV-2 iNOS levels evoked by conditioned media from endothelial cells treated with glucose alone and media derived from endothelial cells exposed to both glucose and hypoxia (Fig. 2b).

### Glucose-Injured Microvascular Endothelial Cells Induce Changes in Inflammatory Proteins Released from BV-2 Cells

The release of cytokines, TNFα and IL-6, by BV-2 cells treated with HBMVEC-conditioned media for 24 h was assessed by ELISA.

**Fig. 3:**
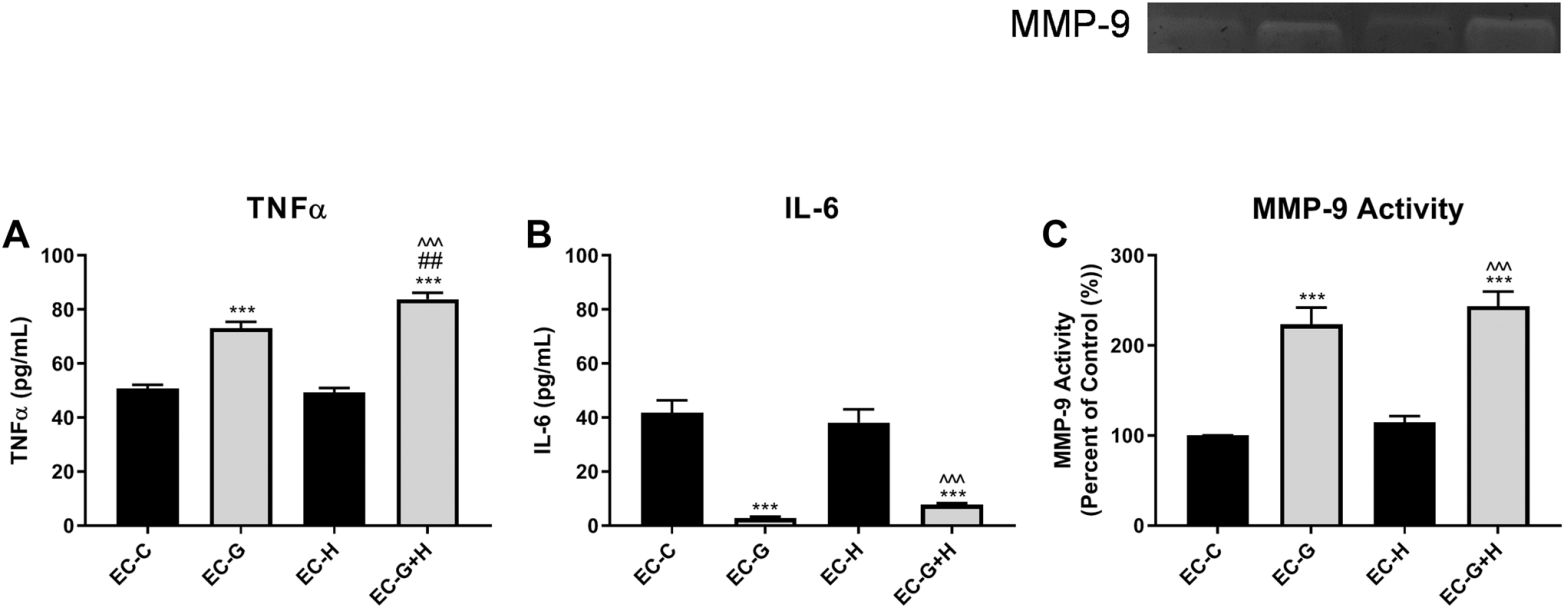
HBMVEC injury induces increased production of inflammatory mediators by BV-2 microglia Conditioned media from injured HBMVECs was used to treat BV-2 microglia for 24 h. Supernatant from BV-2 was collected following treatments. ELISA was used to measure secretion of (**a**) TNFα and (**b**) IL-6. (**c**) The enzymatic activity of MMP-9 was measured by gel zymography (*n* = 3 per group). ***p < 0.001 vs. Control, ^###^*p* < 0.001 vs. Glucose, ^^^*p* < 0.001 vs. Hypoxia.

BV-2 cells that were treated with media from glucose-injured endothelial cells (EC-G) released a significantly (*p* < 0.001) higher level of the pro-inflammatory cytokine TNFα compared to microglial cells exposed to control conditioned media (Fig. 3a). On its own, conditioned media from hypoxic-injured endothelial cells (EC-H) did not alter BV-2 production of TNFα. However, media from endothelial cells that were exposed to both glucose and hypoxia (EC−G+H) increased TNFα production in BV-2 to higher levels than elicited by media from glucose-only injured endothelial cells (*p* < 0.001) (Fig 3a).

In contrast to the results obtained for TNFα, the data showed that conditioned media from glucose-injured endothelial cells (EC-G) caused a significant (*p* < 0.001) decrease in the release of IL-6, a complex cytokine with both pro- and anti-inflammatory activities (Fig. 3b). Conditioned media from hypoxic-injured endothelial cells (EC-H) did not affect BV-2 IL-6 levels. Also, media from endothelial cells exposed to both glucose and hypoxia (EC−G+H) showed a decrease in IL-6 release that was comparable to that observed for glucose alone (EC-G) (Fig. 3b).

Supernatant from BV-2 cells was collected following treatment with HBMVEC-conditioned media for 24 h, as described above, and MMP-9 activity measured by gel zymography. Conditioned media from hypoxia exposed endothelial cells (EC-H) did not affect MMP-9 activity (Fig. 3c). There was a significant (*p* < 0.001) increase in MMP-9 activity, relative to controls, elicited media from glucose only (EC−G) and glucose plus hypoxia (EC−G+H) (Fig. 3c).

### BV-2 Treated with Conditioned Media from Injured Brain Endothelial Cells Show Altered Expression of Microglia Activation Markers

BV-2 cells were treated with HBMVEC-conditioned media for 24 h, as described above, and western blot used to determine changes in protein expression for several microglia activation- and DAM-related markers.

**Fig. 4:**
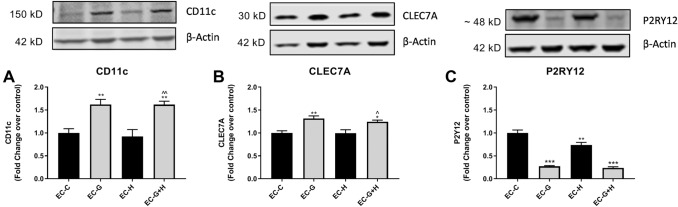
BV-2 treated with conditioned media from injured HBMVEC show altered expression of microglia activation markers Conditioned media from injured HBMVECs was used to treat BV-2 microglia for 24 h. Western blot was used to assess the expression of microglia markers, (**a**) CD11c, (**b**) CLEC7A, and (**c**) P2RY12. *p < 0.05 vs. Control, **p < 0.01 vs. Control, ***p < 0.001 vs. Control, ^*p* < 0.05 vs. Hypoxia, ^^*p* < 0.01 vs. Hypoxia

Changes in the expression of CD11c and CLEC7A by BV-2 in response to endothelial cell conditioned media were similar in pattern, differing slightly in magnitude. Conditioned media from glucose-injured endothelial cells (EC-G) significantly increased expression of CD11c (*p* < 0.01) (Fig. 4a) and CLEC7A (*p* < 0.01) (Fig. 4b) as did conditioned media from glucose plus hypoxia injury (EC-G+H) for both CD11c (*p* < 0.01) and CLEC7A (*p* < 0.05). Exposure of BV-2 cells to HBMVEC-conditioned media collected as described above did not affect expression of TREM2 (data not shown).

Western blot was also used to assess the expression of a homeostatic microglia marker, P2RY12. Expression of P2RY12 was significantly decreased when BV-2 cells were treated with media from glucose injury (EC-G) (*p* < 0.001), hypoxic injury (EC-H) (*p* < 0.01), or glucose plus hypoxic injury (EC-G+H) (*p* < 0.001) compared to controls (Fig. 4c). Together, these changes indicate a shift towards a DAM phenotype in the BV-2 treated with conditioned media from damaged brain endothelial cells.

### The Phagocytic Ability of BV-2 Cells is Altered in Response to Conditioned Media from Injured Endothelial Cells

**Fig. 5:**
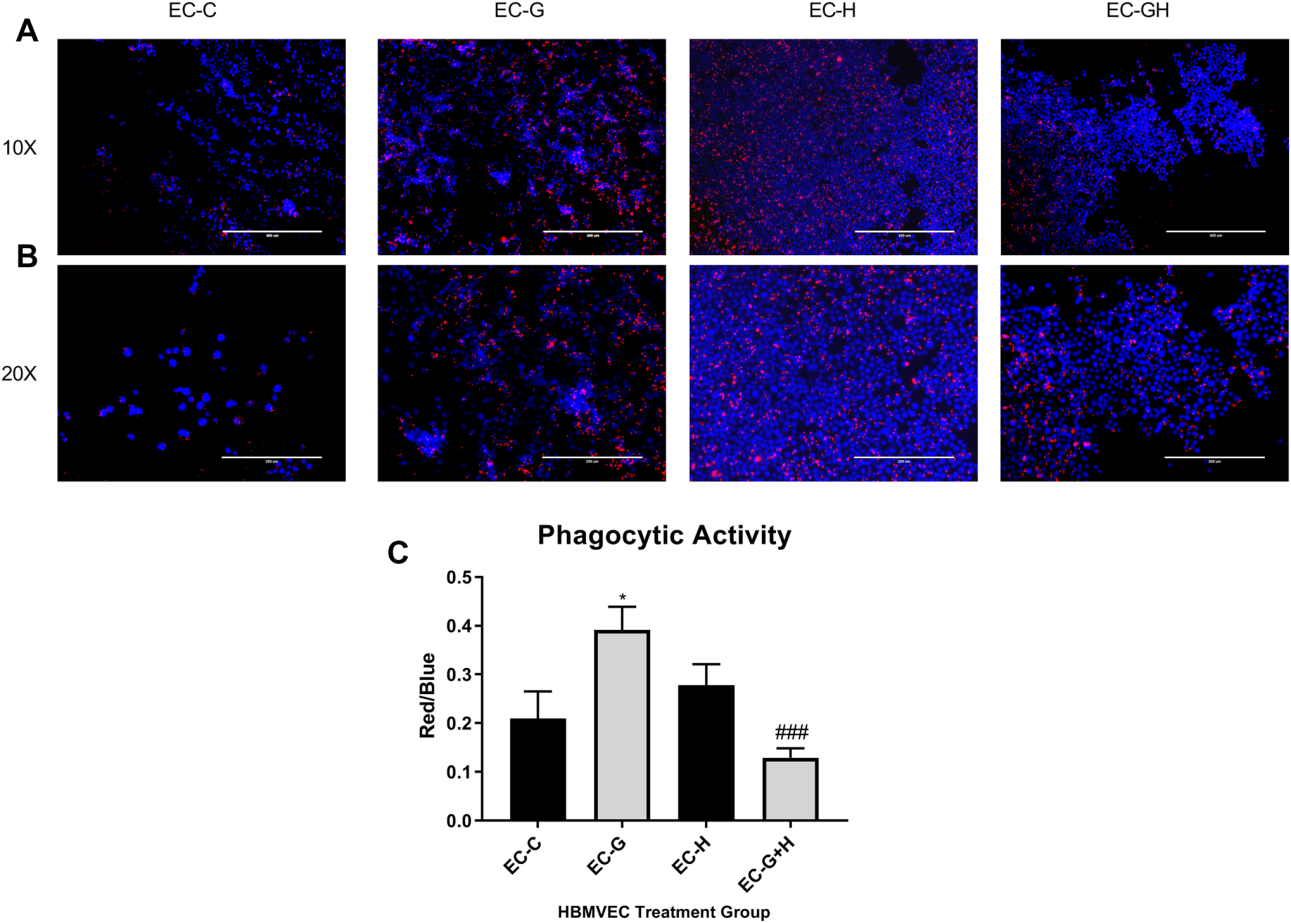
BV-2 treated with conditioned media from injured HBMVEC show altered phagocytic ability Conditioned media from injured HBMVEC was used to treat BV-2 microglia for 24 h. BV-2 were then incubated with fluorescently labeled beads for 1 h. Cells were then imaged (EC-C, EC-G, EC-H, EC-G+H left to right) at 10X (**a**) and 20X (**b**) and bead uptake per cell was quantified (**c**) as a measure of phagocytic activity. *p < 0.05 vs. Control, ^###^*p* < 0.001 vs. Glucose.

BV-2 cells were treated for 24 h with HBMVEC-conditioned media, as described above, and uptake of fluorescently labeled beads measured to assess phagocytic ability (Fig. 5a and b). Bead uptake by BV-2 cells treated with glucose injury media (EC-G) was significantly increased (*p* < 0.05) compared to controls. In contrast, conditioned media from hypoxic injury (EC−H) did not significantly alter phagocytic ability of BV-2 cells. However, treatment of BV-2 with conditioned media from glucose plus hypoxia injury (EC−G+H) significantly (*p* < 0.001) reduced the phagocytic response compared to glucose injury alone conditioned media (EC-G) (Fig. 5c).

## Discussion

The results of this study demonstrate that injured brain endothelial cells can negatively impact the inflammatory/activation state of microglia. These data highlight the importance of cellular cross-talk in the brain, especially among cells in the NVU. The ability of injured endothelial cells to induce changes in microglia, consistent with microglia alterations observed in AD, suggests that this cell-cell interaction is contributory to the development of pathology in AD.

Our data show that brain endothelial cells injured by glucose and hypoxia induce increases in microglial production of NO and TNFα. These inflammatory mediators have been well documented to be elevated in the AD brain in several cell types, including microglia (Hickman et al. 2018). Similarly, our results showing increases in microglial MMP-9 expression by injured endothelial cell conditioned media are consistent with literature documenting elevated MMP-9 in AD (Lorenzl et al. 2003). These results suggest that an injured endothelium may promote the transition of microglia toward an inflammatory phenotype.

Microglia were previously classified dichotomously into M1 and M2 phenotypes using Iba1 as a marker to identify “activated” microglia (Ito et al. 1998). Based on comprehensive gene expression profiling and functional studies, a more fluid spectrum of activation states has been recognized to exist, with the DAM phenotype identified in the AD brain. The DAM phenotype exhibits downregulation of homeostatic microglia genes and upregulation of pro-inflammatory and neurodegeneration-associated genes (Keren-Shaul et al. 2017; Krasemann et al. 2017). Our findings that injured endothelial cells significantly increase microglial expression of DAM-related markers CD11c and CLEC7A, as well as decrease expression of the homeostatic marker P2RY12, suggest conversion of microglia towards a DAM-like phenotype.

Interestingly, treatment of microglial cultures with conditioned media from injured endothelial cells did not affect the expression of TREM2. Although mutations in TREM2 are related to increased AD risk, its precise role is unclear (Jay et al. 2017). One could interpret the lack of effect on TREM2 as a peculiarity related to BV-2 cells and not indicative of microglia in vitro or in vivo. Although there are limitations in using an immortalized cell system to approximate primary cell behavior in vitro, BV-2 cells have been shown to closely model the inflammatory response of primary microglia in vitro (Stansley et al. 2012). In addition, the response of BV-2 cells to LPS has substantial overlap to the response of primary cultured microglia as well as to that of microglia in vivo (Henn et al. 2009). A recent study documented that conversion of microglia to the DAM phenotype occurs in a TREM-independent phase followed by a TREM-dependent phase (Keren-Shaul et al. 2017). It is possible that in our study the transition to the DAM phenotype is not yet complete.

Phagocytosis is a key aspect of microglial function in the healthy brain, responsible for clearance of debris, apoptotic cells, and synaptic pruning (Fu et al. 2014; Hickman et al. 2018). In AD, phagocytic microglia may help to clear aggregated amyloid-β (Aβ) and be beneficial (Fu et al. 2014). In this regard, increased phagocytic activity is correlated with enhanced production of anti-inflammatory and reduced production of pro-inflammatory mediators in microglia and macrophages (Fadok et al. 1998; Wolf et al. 2017; Janda et al. 2018). However, there is still some debate as to whether microglia phagocytosis is beneficial or detrimental in disease. For example, TREM2 mediated phagocytosis of apoptotic neurons is associated with decreased a decreased pro-inflammatory response (Takahashi et al. 2005), while myelin debris phagocytosis enhanced the pro-inflammatory and dampened the anti-inflammatory profile in microglia (Siddiqui et al. 2016). TREM2 knock out mice have impaired phagocytosis, related to increased Aβ plaque burden, reduced microglia localization to plaques, and increased neuritic dystrophy ( Jay et al. 2017; Hickman et al. 2018). DAM exhibit upregulation of phagocytosis-related genes (Keren-Shaul et al. 2017), while other studies indicate that an anti-inflammatory DAM sub-type highly express phagocytosis-associated genes (Rangaraju et al. 2018). Here, the response of BV-2 cells to conditioned media from injured endothelial cells appears to be related to the nature of the endothelial insult. The data show that microglial phagocytosis is increased in response to conditioned media from glucose-injured endothelial cells. In contrast, conditioned media from hypoxia-injured endothelial cells does not significantly alter phagocytosis and further, media from glucose plus hypoxic injured endothelial cells reduces the increase in phagocytosis evoked by glucose alone. These data are similar to the results showing that glucose but not hypoxia increases IL-1β release from endothelial cells and that concurrent hypoxia plus glucose reduces the increase in IL-1β produced by glucose alone. These results support the idea that IL-1β from injured endothelial cells promotes increased phagocytosis by microglia. Other studies have also linked IL-1β to increased microglial phagocytosis as well as to microglial autophagy, a process closely linked to phagocytosis (Ferreira et al. 2011; François et al. 2013; Plaza-Zabala et al. 2017).

Considerable evidence documents an injured cerebrovasculature in AD (Grammas 2011). We have previously shown that damaged brain endothelial cells secrete a number of factors that have been related to neuroinflammation and the activation of pro-inflammatory microglia, including thrombin, MMPs, and a number of cytokines (Grammas and Ovase 2001; Grammas and Ovase 2002; Grammas et al. 2004; Grammas et al. 2011; K Lee et al. 2015; Krasnow et al. 2017). Our findings indicate a role for vascular-derived IL-1β and ROS in propagating the inflammatory cycle in the brain. Here, brain endothelial cells injured by glucose and glucose plus hypoxia exhibit an upregulation in IL-1β and ROS, respectively, and these increases were correlated with the up-regulation of pro-inflammatory markers in microglia. IL-1β acts as an amplifier of immune reactions and can stimulate a number of downstream neuroinflammatory processes. In the periphery, IL-1β stimulated the differentiation of monocytes to M1-like macrophages (Bent et al. 2018). In vitro, IL-1β enhanced microglia release of NO following LPS stimulation (Possel et al. 2000). High levels of oxidative stress have been identified in the AD brain, and ROS function as mediators of injury and inflammation (Fischer and Maier 2015). ROS-mediated signaling pathways are relevant for the induction of inflammatory signaling, including the stimulation of pro-inflammatory cytokines and NO (Fischer and Maier 2015). TNFα, NO, and MMP-9 are all produced in response to both oxidative stress and cytokine signaling (Yong et al. 2001; Clark et al. 2010; Hsieh and Yang 2013; Fischer and Maier 2015), highlighting the potential for these endothelial-derived mediators to activate a pro-inflammatory response in microglia similar to that shown in our in vitro system.

Endothelial cells may react to injurious stimuli such as glucose and hypoxia by activating multiple signaling pathways resulting in the release of diverse mediators (Eelen et al. 2015). In some instances, these mediators could synergize, as we observed for NO release by microglia which was higher when treated with conditioned media from glucose plus hypoxic injury compared to glucose alone. In contrast, the combination of hypoxic and glucose injury reduced the increase in microglial phagocytosis evoked by glucose-only injured endothelial cells. Finally, the responses of cytokines as a family may also vary with individual proteins. In this regard, while TNFα expression by microglia was increased by glucose-injured endothelial cells, IL-6 expression was significantly inhibited. Although mostly regarded as a pro-inflammatory cytokine, IL-6 also has many regenerative or anti-inflammatory activities (Scheller et al. 2011). The pleiotropic actions of IL-6 as both a proinflammatory mediator as well as a neurotrophic factor involved in the physiological homeostasis of the CNS further highlight the complexity of cellular responses in the presence of multiple bioactive moieties.

The relationship between brain endothelial cells and microglia is a growing area of interest, with early findings indicating that damage to brain endothelial cells by a number of mediators can induce an inflammatory response in microglia. Microglia treated with conditioned media from IL-1-treated endothelial cells exhibit increased expression of inflammatory mediators, including IL-1β, IL-6, and CCL2 (Zhu et al. 2019). Treatment of microglia with conditioned media from brain endothelial cells exposed to oxygen-glucose deprivation (OGD) exhibited increases in cytokine release, including TNFα, IL-1β, and IL-10. These microglia additionally exhibited upregulated iNOS expression and impaired phagocytosis (Xing et al. 2018). The OGD-related findings mirror our results in the EC-G and EC-GH microglia treatment groups, particularly with respect to increases in TNFα and iNOS, as well as altered phagocytic ability. Together, these studies further support our hypothesis that the damaged cerebrovasculature can be a driver of inflammation in the brain, and indicate that damage by different mediators may activate diverse inflammatory responses in microglia.

## Conclusions

The results of this study are important as they shed light on the interaction of two cell types, endothelial cells and microglia, likely important in the development of AD pathology. The role of specific mediators in this cell-cell communication is difficult to ascribe because of the inherent limitations of using a simplified in vitro system. Nevertheless, the demonstration that injury to brain endothelial cells by glucose and hypoxia, two insults that are relevant for diabetes, affects processes in microglia that are relevant for AD, support the hypothesis that the cerebral microcirculation could be a nidus where diabetic processes promote the development of AD. A more in-depth investigation of how endothelial cell damage can alter microglia function and lead to an inflammatory response may help us to better understand the neuroinflammation found in AD and other neurodegenerative disorders. Additionally, greater knowledge of the mediators responsible for this endothelial cell-microglia crosstalk could inform novel therapeutic targets and techniques for mitigating disease-related inflammation at its potential point of origin, the vasculature.

## Data Availability

The datasets used and/or analyzed during the current study are available from the corresponding author on reasonable request.

## Abbreviations

AD: Alzheimer’s disease
CVRF: Cardiovascular risk factor
T2D: Type II diabetes mellitus
AGE: Advanced glycation end product
ROS: Reactive oxygen species
CNS: Central nervous system
BBB: Blood-brain barrier
NOX: NADPH oxidase
TNFα: Tumor necrosis factor-α
MMP: Matrix metalloproteinase
MCP-1: Monocyte chemoattractant protein-1
ICAM-1: Intercellular adhesion molecule-1
CAP37: Cationic antimicrobial protein 37 kDa
NO: Nitric oxide
TGF-β: Transforming growth factor-β
IL: Interleukin
NVU: Neurovascular unit
DAM: Disease associated microglia
MGnD: Microglial neurodegenerative phenotype
CR1: Complement receptor 1
TREM2: Triggering receptor expressed on myeloid cells 2
HBMVEC: Human brain microvascular endothelial cell
DMEM: Dulbeccos’s Modified Eagle Medium
Ab/Am: Antibiotic/Antimycotic
FBS: Fetal bovine serum
BSA: Bovine serum albumin
LDH: Lactate dehydrogenase
RT: Room temperature
DCF-DA: 2’,7’-Dichlorofluorescin diacetate
PBS: Phosphate buffered saline
ELISA: Enzyme linked immunosorbent assay
PFA: Paraformaldehyde
ANOVA: Analysis of variance
RNS: Reactive nitrogen species
iNOS: Inducible nitric oxide synthase
Aβ: Amyloid-β
